# Hepatitis B Vaccination Status among Health Care Workers in a Tertiary Hospital in Ethiopia

**DOI:** 10.1155/2017/6470658

**Published:** 2017-06-29

**Authors:** Mohammed Biset Ayalew, Boressa Adugna Horsa

**Affiliations:** Department of Clinical Pharmacy, School of Pharmacy, College of Medicine and Health Sciences, Gondar University, Gondar, Ethiopia

## Abstract

**Background:**

Even though health professionals (HPs) need special consideration for hepatitis B virus (HBV) vaccination, this is not widely implemented in low- and middle-income countries. The aim of this study was to determine the vaccination status of health professionals against hepatitis B virus infection, to identify barriers to vaccination and to identify factors associated with vaccination status.

**Methods:**

This cross-sectional study was conducted from June 1 to 15, 2016. Data was collected using self-administered questionnaire and analysed using SPSS version 21. A test of association was done using binary logistic regression and* P* value less than 0.05 was considered statistically significant.

**Results:**

Out of 286 HPs included in the study 28.7% received full course vaccination against HBV. The most frequently mentioned reasons for not being vaccinated against HBV are unavailability of the vaccine (58.2%) and its cost (18.5%). Age, marital status, level of education, and type of profession have significant association with vaccination status.

**Conclusion:**

HBV coverage among HPs in Gondar University Hospital (GUH) is inadequate. This is mostly due to unavailability of the vaccine and its cost. Vaccination status significantly varies based on level of education, type of profession, age, and marital status. We recommend making vaccines available and accessible.

## 1. Introduction 

Hepatitis B virus (HBV) is highly infectious and causes serious health problems. Worldwide, approximately 2 billion people have been infected with HBV. Of these, 240 million are chronically infected persons with a varying prevalence geographically, highest in Africa and Asia [1, 2]. The major complications of chronic Hepatitis B virus are cirrhosis and hepatocellular carcinoma. An estimated 650,000 people die annually due to chronic Hepatitis B virus infection [3].

HBV is spread primarily by percutaneous or mucosal contact to infected blood and a number of body fluids, including saliva, menstrual, vaginal, and seminal fluids [4]. Transmission of the virus may also result from accidental inoculation of minute amounts of blood or fluid during medical, surgical, and dental procedures or from razors and similar objects contaminated with infected blood; use of inadequately sterilized syringes and needles; intravenous and percutaneous drug abuse; tattooing; body piercing; and acupuncture [3].

Health care providers are at high risk for hepatitis B virus (HBV) infection [5]. The virus remains infectious for prolonged periods on environmental surfaces and is transmissible in the absence of visible blood. Health care providers do not recognize all exposures to potentially infectious blood or body fluids. Health care providers had a prevalence of HBV infection approximately 10 times greater than the general population [6]. The WHO report showed that about 6% of HCPs are each year exposed to blood-borne HBV infections corresponding to about 66,000 HBV infections in health care workers worldwide [7].

Even though there are infection control practices and administration of hepatitis B immune globulin following suspected exposure to reduce the risk of HBV transmission, none have been as effective as active immunization with hepatitis B vaccine, which remains the single most important hepatitis B prevention measure [8, 9].

There is a need to vaccinate all health care providers as a matter of policy and provide postexposure prophylaxis after having significant exposure to patient's blood [10]. Even though health care providers need special consideration for HBV screening and HBV vaccination, this is not widely implemented in low- and middle-income countries [3, 5]. It is the lack of awareness and not giving sufficient attention on part of health care providers coupled with the negligence of the risk that has led them being incompletely vaccinated [11]. The aim of this study was to determine the vaccination status of health professionals against hepatitis B virus infection, to identify barriers for unvaccination, and to identify factors associated with vaccination status.

## 2. Methods

### 2.1. Study Area and Period

This study was done at Gondar University Hospital, Gondar, Ethiopia. Gondar is found in north western Ethiopia, 727 km away from the capital, Addis Ababa. Gondar University Hospital provides service in different departments like gynecology, pediatrics, dentistry, ophthalmology, psychiatry, dermatology, surgery, pharmacy, medical laboratory, and others. It has more than 460 beds. Currently there are 950 health care professionals working in the hospital. The study was conducted from June 1 to June 15, 2016.

### 2.2. Study Design and Subjects

Cross-sectional study design was employed to assess the vaccination status of health professionals against hepatitis B virus infection. All health professionals that were present at work during the study period were considered for the study. Health professionals who were not voluntary to participate were excluded. Sample size was calculated using single population proportion formula assuming the prevalence of HBV vaccination among health professionals as 50% in order to get larger sample size. Correction formula was used because the source population was less than 10,000. Finally 286 HPs were included in the study. Study participants were selected by using simple random sampling technique/lottery method from the list of health works available during the study period.

### 2.3. Data Collection and Management

Structured data collection format was developed by the research authors after reviewing literatures done on the topic in various parts of the world [12–14]. The data collection tool consists of participant's sociodemographic characteristics, questions that measure vaccination status of participants, and barriers for vaccination. In order to check any inconsistency the data collection tool was pretested on 5% of the sample population and necessary adjustments were done before the actual period of data collection. Data collection was done by two graduating pharmacy students by distributing self-administered questionnaire prepared in English language to the participants and recollecting it. The principal investigator cheeked the completeness, consistency, and accuracy of the collected data every day.

### 2.4. Data Analysis

Data was coded and entered into the Epinfo (version 7) software. Then, data was exported to SPSS (version 21) for analysis. Descriptive statistics was done and results were presented in tables. A test of association was done using binary logistic regression. All the statistical tests were significant at *P* value less than 0.05.

### 2.5. Ethical Consideration

Ethical clearance was obtained from the Ethical Review Committee of School of Pharmacy, University of Gondar. Medical director of the hospital and other concerned officials were communicated and permission was granted. The respondents were informed about the purpose of the study. Confidentiality of the information taken was assured; thus the name and address of the participants were not recorded in the data collection format.

## 3. Results

### 3.1. Sociodemographic Characteristics of Health Professionals

As shown in Table 1 out of 286 HPs included in the study, 186 (55.6%) were males. Majority of HPs (82.9%) were in the age group of 21–29 years and mean age of the HPs was 26.91 ± 4.57 years (range 21–58). About two-thirds (67.1%) were unmarried and more than three-fourths (76.9%) were orthodox religion followers. Most of the HPs were nurses (32.9%) and medical doctors (31.8%). About three-fourths (75.2%) of the HPs had less than five years of work experience.

As indicated in Table 2 more than half (51%) of health professionals have never received hepatitis B vaccine. From health professionals who received the vaccine only 82 (58.6%) received the full course of the vaccine. Overall percentage of HPs in GUH who receive full course vaccination against HBV is 28.7%.

The most frequently mentioned reasons for not being vaccinated against HBV are unavailability of the vaccine (58.2%) and its cost (18.5%). Being busy is the common reason (32.8%) for not receiving full course of vaccination. As shown in Table 3 about one-fourth (25.9%) of the study participants started the vaccination and wait for the next dose.

The result of multivariate binary logistic regression analysis showed that age, marital status, level of education, and type of profession have significant association with vaccination status.

As indicated in Table 4 unmarried health professionals, first degree holders, master degree holders, medical doctors, and pharmacists have better vaccination status than others. Professionals with age of 30–39 years have 11.1 times lower vaccination status than those health professionals with age 40 years and above.

As shown in Table 5 from all of the sociodemographic characteristics of health professionals only the type of profession has significant association with completion of the full course of HBV vaccination. Accordingly laboratory technologists are 12.5 times less likely to complete their HBV vaccination.

## 4. Discussion

Although hepatitis B is preventable disease it is one of the major causes of morbidity and mortality throughout the world including Ethiopia. Prevention is ultimately the most efficient means towards improved health [15]. Immunization programs are highly effective and clearly protect populations and individuals at risk if they are properly implemented [16]. According to WHO estimate, hepatitis B vaccination coverage among health care workers varies from 18% being lowest in Africa to highest 77% in Australia and New Zealand [7].

In this study less than half (49%) of health professionals working in Gondar University Hospital were received hepatitis B virus vaccination. A very similar result was reported in studies conducted in Burkina Faso, Japan, and India which indicated vaccination coverage of 47.7%, 48.2%, and 51.2%, respectively [17–19]. But this is lower vaccination coverage as compared to what was reported in studies conducted in Nigeria (91.9%), Sweden (79%), Pakistan (76%), and India (79.7 and 70.3%) [20–24]. The fact that most of the respondents in the current study were young and had fewer years of practice may be responsible for the low rate of vaccination as it is found in this study that younger age is significantly associated with unvaccination.

From those health professionals who reported that they are vaccinated only 58.6% of them received the full course of the vaccine. Overall only 28.7% of the health professionals in GUH have received full dose of HBV vaccine. This is a lower level of complete vaccination when compared with the results of other previous studies. Studies conducted in USA and France reported that 75% and 93% of health care workers were completely vaccinated [22, 23]. There are also several studies that reported around half (46.2%–57.6%) of health care workers were completely vaccinated for HBV [19, 23–26]. The lowest rate of complete vaccination in this study therefore means that health professionals should not only be encouraged to receive hepatitis B vaccine but also be encouraged to complete the dose to ensure the effectiveness of this vaccine. Those with incomplete vaccination status should also be encouraged to have antihepatitis B titers measured to determine their level of protection and if there will be a need for a further dose of the vaccine.

For health professionals who did not take the vaccine, the frequently stated reasons were unavailability of the vaccine and its price each comprising 58.2% and 18.5%, respectively. Similarly unavailability of the vaccine is mentioned in different studies as one of the main reasons for not being vaccinated [12, 19, 21, 27]. Studies conducted in Bahir Dar, Iran, and Addis Ababa also indicated that the cost of the vaccine was a barrier for vaccination in addition to vaccine unavailability [12, 19, 27].

Being busy is the common reason (32.8%) for not completing the vaccination. In line with this Chaudhari et al. reported that absence during vaccine drive due to leave or temporary duty was one of the most common reasons for not completing primary immunization in partially vaccinated health care workers [28]. In the current study about one-fourth (25.9%) of partially vaccinated health professionals were on waiting for the next dose. According to the study done in selected public hospitals of Addis Ababa for study participants who did not complete the vaccine series, all of them said that they were on wait for the last dose [12].

The findings of this study revealed that the type of profession, level of education, participants age, and marital status have significant association with vaccination status. Significantly higher vaccination coverage was found for medical doctors, pharmacists, first degree holders, master degree holders, and unmarried health professionals than their equivalents in the group. The presence of significant association between type of profession and vaccination status is also reported by so many studies [12, 13, 25, 28, 29]. The study done by Kathy et al. reported that more than high school education has significantly higher vaccination coverage [14]. Younger health professionals have less vaccination coverage than those who are forty years old and above. This may be due to young professionals stay in the institution is relatively less and have less chance of getting vaccination campaign programmes as compared to those who stayed longer in the institution. Similarly Olubuyide et al. in a study among Nigerian doctors and dentists reported that unvaccinated personnel were more likely to be surgeons or dentists less than 37 years of age [30].

## 5. Limitation

The result of the study cannot be generalized as it is a single centered study. Vaccination status was self-reported and not verified by vaccination records. So recall bias could have led to over- or underestimation of coverage.

## 6. Conclusion

We observed a generally low rate of HBV vaccine coverage among health professionals in Gondar University Hospital. Vaccination status significantly varies based on level of education, type of profession, age, and marital status. Unavailability and cost of the vaccine were the major barriers for unvaccination. Continued efforts are needed to increase the vaccine coverage among unvaccinated health professionals to protect workers and patients.

## Figures and Tables

**Table 1:** Sociodemographic characteristics of health professionals, GUH, Ethiopia, 2016.

Characteristics	Frequency (*n*)	Percentage (%)
Sex		
Male	181	63.3
Female	105	36.7
Age (yrs)		
21–29	237	82.9
30–39	41	14.3
>40	8	2.8
Religion		
Orthodox Christians	220	76.9
Muslim	28	9.8
Protestant Christians	29	10.1
Others^a^	9	3.1
Marital status		
Unmarried	192	67.1
Married	94	32.9
Education level		
Certificate	6	2.1
Diploma	9	3.1
First degree	241	84.3
Master degree	21	7.3
Specialist	9	3.1
Profession		
Medical doctors	91	31.8
Nurses	94	32.9
Health officers	6	2.1
Pharmacist	34	11.9
Lab. technologists	26	9.1
Midwives	20	7
Others^b^	15	5.2
Work experience		
<5 years	215	75.2
≥5 years	71	24.8
Department		
Surgical department	35	12.2
Laboratory	28	9.8
Delivery unit	27	9.4
Emergency department	15	5.2
Internal medicine	75	26.2
Pediatrics	53	18.5
Pharmacy unit	15	5.2
Gynecology/obstetrics	13	4.5
Others^c^	25	8.7

^a^Catholic, atheist, and Hindu; ^b^health officer, anesthesia, dentist, physiotherapies, and psychiatry nurse; ^c^dental department, oncology, optometry, psychiatry ward, recovery, and general ward.

**Table 2:** Vaccination status of health professionals against HBV infection, GUH, Ethiopia, 2016.

Characteristics	Frequency (*n*)	Percentage (%)
Ever received hepatitis B vaccination	140	49
Never received hepatitis B vaccination	146	51
total	286	100

Received full doses of vaccine	82	58.6
Did not receive full doses of vaccine	58	41.4
Total	140	100

**Table 3:** Reasons for not being vaccinated against HBV and for not taking full course of the vaccine, GUH, Ethiopia, 2016.

	Reasons	Frequency (*n*)	Percentage (%)
Reason for not being vaccinated (*n* = 146)	Cost of the vaccine	27	18.5
	The vaccine is not easily available	85	58.2
	Afraid of vaccine side effect	3	2.1
	Vaccination is not necessary	2	1.4
	Lack of information	12	8.2
	Too busy	12	8.2
	Afraid of needles	1	0.7
	Others^d^	4	2.7

Reason for not receiving full course of vaccination (*n* = 58)	Fear of adverse effect	10	17.2
	Assuming that it was enough	2	3.4
	Being busy	19	32.8
	Not available	8	13.8
	Carelessness	4	6.9
	Started and waited	15	25.9

^d^HBV positive, carelessness, and not useful.

**Table 4:** Factors associated with the HBV vaccination status of health professionals in GUH, Ethiopia, 2016.

Characteristics	Yes (%)	No (%)	COR (95% CI)	AOR (95% CI)
Sex				
Male	74 (52.9%)	107 (73.3%)	2.45 (1.49–4.01)^*∗*^	1.07 (0.52–2.18)
Female	66 (47.1%)	39 (26.7%)	1.00	1.00
Age** (**yrs)				
21–29	107 (76.4%)	130 (89%)	0.73 (0.17–3.12)	0.22 (0.03–1.45)
30–39	30 (21.4%)	11 (7.5%)	0.22 (0.05–1.08)	0.09 (0.01–0.65)^*∗*^
≥40	3 (2.1%)	5 (3.4%)	1.00	1.00
Religion				
Orthodox	109 (77.9%)	111 (76.0%)	0.51 (0.12–2.09)	0.29 (0.05–1.81)
Muslim	16 (11.4%)	12 (8.2%)	0.38 (0.08–1.81)	0.24 (0.03–1.77)
Protestant	12 (8.6%)	17 (11.6%)	0.71 (0.15–3.41)	0.21 (0.03–1.66)
Others^a^	3 (2.1%)	6 (4.1%)	1.00	1.00
Marital status				
Unmarried	72 (51.4%)	120 (82.2%)	4.36 (2.55–7.47)^*∗*^	2.93 (1.34–6.44)^*∗*^
Married	68 (48.6%)	26 (17.8%)	1.00	1.00
Education level				
Certificate	4 (2.9%)	2 (1.4%)	0.63 (0.07–5.35)	4.20 (0.28–62.67)
Diploma	4 (2.9%)	5 (3.4%)	1.56 (0.24–10.03)	8.25 (0.63–107.90)
First degree	114 (81.4%)	127 (87.0%)	1.39 (0.37–5.31)	7.61 (1.18–49.26)^*∗*^
Master degree	13 (9.3%)	8 (5.5%)	0.77 (0.18–3.74)	11.59 (1.34–100.48)^*∗*^
Specialist	5 (3.6%)	4 (2.7%)	1.00	1.00
Profession				
Medical doctor	15 (10.7%)	76 (52.1%)	13.93 (3.91–49.68)^*∗*^	20.60 (3.38–125.68)^*∗*^
Nurse	62 (44.3%)	32 (21.9%)	1.42 (0.42–4.81)	1.83 (0.34–9.68)
Health officer	4 (2.9%)	2 (1.4%)	1.38 (0.18–10.65)	1.59 (0.13–18.86)
Pharmacist	15 (10.7%)	19 (13.0%)	3.48 (0.92–13.17)	9.64 (1.43–65.17)^*∗*^
Lab. technologist	22 (15.7%)	4 (2.7%)	0.50 (0.11–2.39)	0.00
Midwifes	11 (7.9%)	9 (6.2%)	2.25 (0.53–9.54)	2.92 (0.44–19.31)
Others^b^	11 (7.9%)	4 (2.7%)	1.00	1.00
Work experience				
<5 years	98 (70.0%)	117 (80.1%)	1.73 (1.00–2.98)^*∗*^	0.51 (0.22–1.18)
≥5 years	42 (30.0%)	29 (19.9%)	1.00	1.00
Department				
Surgery	15 (10.7%)	20 (13.7%)	2.833 (0.968–8.297)	1.20 (0.29–5.07)
Laboratory	22 (15.7%)	6 (4.1%)	0.580 (0.169–1.989)	1.35 (0.31–14.41)
Delivery unit	11 (7.9%)	16 (11.0%)	3.091 (0.990–9.647)	1.68 (0.36–7.84)
Emergency	8 (5.7%)	7 (4.8%)	1.859 (0.498–6.941)	1.11 (0.21–5.98)
Internal medicine	35 (25.0%)	40 (27.4%)	2.429 (0.935–6.311)	0.70 (0.19–2.57)
Pediatrics	16 (11.4%)	37 (25.3%)	4.914 (1.764–13.692)^*∗*^	1.76 (0.44–7.03)
Pharmacy	9 (6.4%)	6 (4.1%)	1.417 (0.374–5.365)	0.36 (0.06–2.29)
Gyn./obs.	7 (5.0%)	6 (4.1%)	1.821 (0.460–7.216)	0.52 (0.09–3.05)
Others^c^	17 (12.1%)	8 (5.5%)	1.00	1.00

^a^Catholic, atheist, and Hindu; ^b^health officer, anesthesia, dentist, and physiotherapies; ^c^dental department, oncology, optometry, psychiatry ward, recovery ward, and general ward. *∗* indicates statistically significant value.

**Table 5:** Factors associated with the completion of full course HBV vaccination among health professionals who took at least 1 dose of the vaccine in GUH, Ethiopia, 2016.

Characteristics	Yes (%)	No (%)	COR (95% CI)	AOR (95% CI)
Sex				
Male	39 (47.6%)	35 (60.3%)	1.68 (0.85–3.32)	1.61 (0.65–4.03)
Female	43 (52.4%)	23 (39.7%)	1.00	1.00
Age (yrs)				
21–29	65 (79.3%)	42 (72.4%)	0.32 (0.03–3.68)	0.53 (0.03–8.86)
30–39	16 (19.5%)	14 (24.1%)	0.44 (0.04–5.36)	0.73 (0.04–12.53)
>40	1 (1.2%)	2 (3.4%)	1.00	1.00
Religion				
Orthodox	70 (85.4%)	39 (67.2%)	0.28 (0.02–3.17)	0.54 (0.03–11.04)
Muslim	9 (11.0%)	7 (12.1%)	0.39 (0.03–5.21)	0.44 (0.02–10.28)
Protestant	2 (2.4%)	10 (17.2%)	2.50 (0.15–42.80)	7.59 (0.25–231.59)
Others^a^	1 (1.2%)	2 (3.4%)	1.00	1.00
Marital status				
Unmarried	40 (48.8%)	32 (55.2%)	1.29 (0.66–2.54)	1.46 (0.56–3.83)
Married	42 (51.2%)	26 (44.8%)	1.00	1.00
Education level				
Certificate	2 (2.4%)	2 (3.4%)	0.67 (0.05–9.47)	2.36 (0.06–86.69)
Diploma	2 (2.4%)	2 (3.4%)	0.67 (0.05–9.47)	7.57 (0.19–301.00)
First degree	67 (81.7%)	47 (81.0%)	0.47 (0.08–2.91)	1.86 (0.11–31.12)
Master degree	9 (11.0%)	4 (6.9%)	0.30 (0.04–2.52)	0.60 (0.03–13.60)
Specialist	2 (2.4%)	3 (5.2%)	1.00	1.00
Profession				
Medical doctor	7 (8.5%)	8 (13.8%)	0.65 (0.13–3.21)	0.31 (0.03–3.17)
Nurse	38 (46.3%)	24 (41.4%)	0.36 (0.10–1.37)	0.31 (0.05–2.13)
Health officer	2 (2.4%)	2 (3.4%)	0.57 (0.06–5.78)	0.23 (0.01–4.50)
Pharmacist	6 (7.3%)	9 (15.5%)	0.86 (0.17–4.27)	1.87 (0.16–22.11)
Lab. technologist	18 (22.0%)	4 (6.9%)	0.13 (0.03–0.65)	0.08 (0.01–0.63)^*∗*^
Midwifes	7 (8.5%)	4 (6.9%)	0.33 (0.06–1.86)	0.71 (0.06–9.05)
Others^b^	4 (4.9%)	7 (12.1%)	1.00	1.00
Work experience				
<5 years	59 (72.0%)	39 (70.0%)	0.80 (0.39–1.66)	0.71 (0.25–2.04)
≥5 years	23 (28.0%)	42 (30.0%)	1.00	1.00
Department				
Surgery	8 (9.8%)	7 (12.1%)	0.98 (0.25–3.96)	0.98 (0.17–5.71)
Laboratory	18 (22%)	4 (6.9%)	0.25 (0.06–1.06)	0.47 (0.06–3.92)
Delivery	7 (8.5%)	4 (6.9%)	0.64 (0.14–3.04)	0.19 (0.02–2.27)
Emergency	4 (4.9%)	4 (6.9%)	1.13 (0.21–6.05)	0.71 (0.08–6.61)
Internal medicine	16 (19.5%)	19 (32.8%)	1.34 (0.42–4.27)	1.06 (0.23–4.92)
Pediatrics	10 (12.2%)	6 (10.3%)	0.68 (0.17–2.71)	0.84 (0.15–4.85)
Pharmacy	6 (7.3%)	3 (5.2%)	0.56 (0.11–3.02)	0.10 (0.01–1.77)
Gynecology/obstetrics	4 (4.9%)	3 (5.2%)	0.84 (0.14–4.97)	0.55 (0.06–5.21)
Others^c^	9 (11.0%)	8 (13.8%)	1.00	1.00

^a^Catholic, atheist, and Hindu; ^b^health officer, anesthesia, dentist, and physiotherapies; ^c^dental department, oncology, optometry, psychiatry ward, recovery ward, and general ward. *∗* indicates statistically significant value.

## Data Access

The datasets analysed during the current study available from the corresponding author on reasonable request.
